# Multilocus Microsatellite Typing (MLMT) of Strains from Turkey and Cyprus Reveals a Novel Monophyletic *L. donovani* Sensu Lato Group

**DOI:** 10.1371/journal.pntd.0001507

**Published:** 2012-02-14

**Authors:** Evi Gouzelou, Christos Haralambous, Ahmad Amro, Andreas Mentis, Francine Pratlong, Jean-Pierre Dedet, Jan Votypka, Petr Volf, Seray Ozensoy Toz, Katrin Kuhls, Gabriele Schönian, Ketty Soteriadou

**Affiliations:** 1 Laboratory of Molecular Parasitology, Hellenic Pasteur Institute, Athens, Greece; 2 Faculty of Pharmacy, Al-Quds University, Jerusalem, Palestine; 3 Laboratory of Medical Microbiology, Hellenic Pasteur Institute, Athens, Greece; 4 Laboratoire de Parasitologie and Centre National de Référence des Leishmania, Université Montpellier 1 and CHU Montpellier, Montpellier, France; 5 Department of Parasitology, Faculty of Science, Charles University, Prague, Czech Republic; 6 Department of Parasitology, Ege University Medical School, Bornova, Izmir, Turkey; 7 Institut für Mikrobiologie und Hygiene, Charité Universitätsmedizin Berlin, Berlin, Germany; Lancaster University, United Kingdom

## Abstract

**Background:**

New foci of human CL caused by strains of the *Leishmania donovani* (*L. donovani*) complex have been recently described in Cyprus and the Çukurova region in Turkey (*L. infantum*) situated 150 km north of Cyprus. Cypriot strains were typed by Multilocus Enzyme Electrophoresis (MLEE) using the Montpellier (MON) system as *L. donovani* zymodeme MON-37. However, multilocus microsatellite typing (MLMT) has shown that this zymodeme is paraphyletic; composed of distantly related genetic subgroups of different geographical origin. Consequently the origin of the Cypriot strains remained enigmatic.

**Methodology/Principal Findings:**

The Cypriot strains were compared with a set of Turkish isolates obtained from a CL patient and sand fly vectors in south-east Turkey (Çukurova region; CUK strains) and from a VL patient in the south-west (Kuşadasi; EP59 strain). These Turkish strains were initially analyzed using the *K26*-PCR assay that discriminates MON-1 strains by their amplicon size. In line with previous DNA-based data, the strains were inferred to the *L. donovani* complex and characterized as non MON-1. For these strains MLEE typing revealed two novel zymodemes; *L. donovani* MON-309 (CUK strains) and MON-308 (EP59). A population genetic analysis of the Turkish isolates was performed using 14 hyper-variable microsatellite loci. The genotypic profiles of 68 previously analyzed *L. donovani* complex strains from major endemic regions were included for comparison. Population structures were inferred by combination of Bayesian model-based and distance-based approaches. MLMT placed the Turkish and Cypriot strains in a subclade of a newly discovered, genetically distinct *L. infantum* monophyletic group, suggesting that the Cypriot strains may originate from Turkey.

**Conclusion:**

The discovery of a genetically distinct *L. infantum* monophyletic group in the south-eastern Mediterranean stresses the importance of species genetic characterization towards better understanding, monitoring and controlling the spread of leishmaniasis in this region.

## Author Summary

In eastern Mediterranean, leishmaniasis represents a major public health problem with considerable impact on morbidity and potential to spread. Cutaneous leishmaniasis (CL) caused by *L. major* or *L. tropica* accounts for most cases in this region although visceral leishmaniasis (VL) caused by *L. infantum* is also common. New foci of human CL caused by *L. donovani* complex strains were recently described in Cyprus and Turkey. Herein we analyzed Turkish strains from human CL foci in Çukurova region (north of Cyprus) and a human VL case in Kuşadasi. These were compared to Cypriot strains that were previously typed by Multilocus Enzyme Electrophoresis (MLEE) as *L. donovani* MON-37. Nevertheless, they were found genetically distinct from MON-37 strains of other regions and therefore their origin remained enigmatic. A population study was performed by Multilocus Microsatellite Typing (MLMT) and the profile of the Turkish strains was compared to previously analyzed *L. donovani* complex strains. Our results revealed close genetic relationship between Turkish and Cypriot strains, which form a genetically distinct *L. infantum* monophyletic group, suggesting that Cypriot strains may originate from Turkey. Our analysis indicates that the epidemiology of leishmaniasis in this region is more complicated than originally thought.

## Introduction

Leishmaniases are a group of neglected infectious diseases caused by obligate intracellular protozoa of the genus *Leishmania* and transmitted by sand flies of the Phlebotominae subfamily. They are characterized by a spectrum of clinical manifestations ranging from ulcerative skin lesions (cutaneous leishmaniasis, CL) to a life-threatening disseminated visceral infection (visceral leishmaniasis, VL). The overall prevalence of leishmaniasis is estimated to 12 million cases worldwide, and the global yearly incidence of all clinical forms is two million cases [1].

In the Old World, *Leishmania major* and *L. tropica* are the prevalent dermotropic species causing CL, whereas strains belonging to the *L. donovani* complex are typically responsible for VL. The current nomenclature of this complex encompasses only two species, *L. donovani* and *L. infantum*. *L. archibaldi* previously described for strains isolated in East African VL foci has been shown to be an invalid species [2], and *L. chagasi* was found to be virtually identical to *L. infantum* from Southern Europe and recently introduced to the Americas [3]. Some *L. infantum* variants found in Mediterranean countries are predominantly dermotropic [4], [5], [6].

In the Eastern Mediterranean Region (EMR) leishmaniasis represents a major public health problem with considerable impact on morbidity and the potential to spread. Zoonotic and anthroponotic CL caused by *L. major* and *L. tropica*, respectively account for the largest number of cases in this region, although zoonotic VL caused by *L. infantum* is also common. Nevertheless, data on causative agents, vectors or reservoirs are not regularly available [7].

In this context, Turkey a country that lies at the crossroad between Asia and Europe represents a geographic site of special epidemiological interest regarding leishmaniasis. Both zoonotic VL caused by *L. infantum* and anthroponotic CL due to *L. tropica* have long been known to exist in several regions of Turkey [8]. VL is endemic throughout the Aegean, Central Anatolia, Marmara, Mediterranean and Black Sea Regions and CL is hyperendemic in south-eastern parts of Turkey neighbouring Iraq and Syria. According to Multilocus Enzyme Electrophoresis (MLEE), which remains the reference method for typing *Leishmania* strains [1], *L. infantum* MON-1 is the prevalent zymodeme causing VL in Turkey, as in all Mediterranean countries. CL cases caused by *L. infantum* spp. are mainly reported from the Çukurova region of south-east Turkey. In particular, Multilocus Sequence Typing (MLST) followed by phylogenetic analysis assigned a single strain isolated from a CL human case and two *Phlebotomus tobbi* (*P. tobbi*) sand fly isolates from the Çukurova region in Adana province (CUK1, CUK2 and CUK10) to *L. infantum*. However, the isolates did not group with the MON-1 clade but with a MON-188 strain (ISS800) from Italy (Sicily) [6].

CL cases due to species of the *L. donovani* complex have also been reported in adjacent areas of Middle East countries, such as Lebanon and Syria, where the causative agents were identified as *L. infantum* by MLEE typing [9]. In another study a CL isolate from Lebanon was characterized as *L. archibaldi* [10]. Also, it was shown that both *L. infantum* and *L. donovani* parasites circulate in the northwest of Iran [11]. Notably, CL cases due to *L. donovani* MON-37 were also detected in Cyprus [12], [13].

Interestingly, most reported CL cases including those from Turkey fall within a 500 km radius area. All have been typed at species level mostly by PCR-based molecular methods, which for practical reasons have replaced MLEE. It is therefore possible that in the past, due to the lack of easily applicable *Leishmania* PCR-typing tools, some CL cases identified in this region were falsely attributed to the traditional dermotropic species (*L. major*, *L. tropica*) based solely on clinical criteria. Hence, strains of the *L. donovani* complex could potentially have a substantial contribution to the generation of CL in the EMR.

All the above point to the need for an in-depth study of the genetic diversity of parasite populations in the region, at different levels (genus, complex, species, population or even strain). This can be achieved using methods with high discriminatory power and reproducibility that allow inter-lab comparisons [14], [15] such as multilocus microsatellite typing (MLMT). Notably, MLMT has been extensively used for population genetic studies in *Leishmania* throughout the world giving useful insights into the epidemiology of leishmaniasis [3], [16], [17], [18]. This approach can contribute to a better understanding of the geographical distribution and dynamics of *Leishmania* populations and disease epidemiology in the EMR.

In this context, we have analyzed a set of *L. donovani* complex strains isolated from human CL foci in Çukurova and from a VL patient from Kuşadasi in the Aegean region of Turkey. This set was compared to strains from Cyprus causing VL or CL that were MLEE- typed as *L. donovani* MON-37 [12] but shown by MLMT to be genetically distinct from MON-37 strains of other regions [16]. The strains were first analyzed using the *K26*-assay that discriminates *L. donovani* complex subspecies and then typed further using MLEE. In order to elucidate their population structure, the MLMT approach was applied. The microsatellite profiles of 68 previously analyzed *Leishmania donovani* complex strains from major endemic regions of VL, including those from Cyprus, were used in the analysis for comparison.

## Materials and Methods

### *Leishmania* strains, MLEE and DNA extraction

Table 1 lists the 76 *L. infantum* and *L. donovani* strains used in this study along with their WHO code, geographic origin, zymodeme type, pathology and population assignment. The *Leishmania* strains from Turkey included EP59 [19] isolated from a VL patient in Kuşadasi province in the Aegean region of Turkey (nearby Izmir) and six strains from Çukurova region in south-eastern Turkey [6]. The latter were five isolates from *P. tobbi* (CUK2, CUK3, CUK4, CUK7, CUK10) and a single isolate from a human CL case (CUK1). We have also included three MON-37 clones of CD44, isolated in 2005 from a dog in Cyprus. Strains EP59, CUK1, CUK2 and CUK3 were typed by MLEE using the Montpellier method (MON), as described by Rioux et al. [20].

**Table 1:** Characteristics of the 76 *Leishmania* strains used in this study.

*Strain code (lab code)*	*WHO Code*	*Country/Region*	*Zymodeme*	*Pathology*	*Population assignment*
LEM3249 (LG7)	MHOM/FR/96/LEM3249	France/Pyrénées-Orientales	MON-29	CL	*L. infantum* non MON-1 (Pop. 5A)
LEM2298 (LG8)	MHOM/ES/91/LEM2298	Spain/Valencia	MON-183	VL	*L. infantum* non MON-1 (Pop. 5A)
LEM189 (LG14)	MHOM/FR/80/LEM189	France/Pyrénées-Orientales	MON-11	CL	*L. infantum* non MON-1 (Pop. 5A)
LLM175 (LG19)	MHOM/ES/88/LLM175	Spain/Madrid	MON-198	VL	*L. infantum* non MON-1 (Pop. 5A)
LLM373 (LG20)	MHOM/ES/92/LLM373	Spain/Madrid	MON-199	VL	*L. infantum* non MON-1 (Pop. 5A)
ISS1036 (LG21)	MHOM/IT/94/ISS1036	Italy	MON-228	VL	*L. infantum* non MON-1 (Pop. 5A)
ISS800 (LG22)	MHOM/IT/93/ISS800	Italy/Sicily	MON-188	VL	*L. infantum* non MON-1 (Pop. 5B)
BUCK (LG15)	MHOM/MT/85/BUCK	Malta	MON-78	CL	*L. infantum* non MON-1 (Pop. 5B)
LLM-707	MHOM/ES/97/LLM-707	Spain/Madrid	MON-24	VL (HIV+)	*L. infantum* non MON-1 (Pop. 4)
LLM-1036	MHOM/ES/2001/LLM-1036	Spain/Madrid	MON-27	VL (HIV+)	*L. infantum* non MON-1 (Pop. 4)
LLM-745	MHOM/ES/98/LLM-745	Spain/Andalucia	MON-34	VL (HIV+)	*L. infantum* non MON-1 (Pop. 4)
IMT238	MHOM/PT/98/IMT238	Portugal/Lisbon-MRL	MON-80	VL (HIV+)	*L. infantum* non MON-1 (Pop. 4)
CH32	MHOM/CY/2006/CH32	Cyprus/Paphos	MON-37	CL	CY non MON-1
CH33	MHOM/CY/2006/CH33	Cyprus/Paphos	MON-37	VL	CY non MON-1
CH34	MHOM/CY/2006/CH34	Cyprus/Paphos	MON-37	CL	CY non MON-1
CH35	MHOM/CY/2006/CH35	Cyprus/Paphos	MON-37	CL	CY non MON-1
CH36	MHOM/CY/2006/CH36	Cyprus/Limassol	MON-37	VL	CY non MON-1
**CD44 cl.1** *	**MCAN/CY/2005/CD44 cl.1**	**Cyprus/Paphos**	**MON-37**	**CanL**	**CY non MON-1**
**EP59**	**MHOM/TR/2001/EP59**	**Turkey/Kuşadası**	**MON-308**	**VL**	**TR non MON-1 & GR/TR MON-1** **
**CUK1**	**MHOM/TR/2005/CUK1**	**Turkey/Çukurova**	**MON-309**	**CL**	**TR non MON-1**
**CUK2**	**ITOB/TR/2005/CUK2**	**Turkey/Çukurova**	**MON-309**	**Sandfly**	**TR non MON-1**
**CUK3**	**ITOB/TR/2005/CUK3**	**Turkey/Çukurova**	**n.d.**	**Sandfly**	**TR non MON-1**
**CUK4**	**ITOB/TR/2006/CUK4**	**Turkey/Çukurova**	**n.d.**	**Sandfly**	**TR non MON-1**
**CUK7**	**ITOB/TR/2006/CUK7**	**Turkey/Çukurova**	**n.d.**	**Sandfly**	**TR non MON-1**
**CUK10**	**ITOB/TR/2005/CUK10**	**Turkey/Çukurova**	**MON-309**	**Sandfly**	**TR non MON-1**
GEBRE 1 (LG11)	MHOM/ET/72/GEBRE 1	Ethiopia	MON-82	VL	SD/ET2
GILANI (LG12)	MHOM/SD/82/GILANI	Sudan	MON-30	VL	SD/ET2
3S (LG18)	MHOM/SD/62/3S	Sudan	MON-81	VL	SD/ET2
HUSSEN (LG13)	MHOM/ET/00/HUSSEN	Ethiopia	MON-31	VL	SD/ET1
LEM3946 (LG17)	MCAN/SD/2000/LEM3946	Sudan/Gedaref	MON-274	CanL	SD/ET1
LEM3472 (LG23)	MHOM/SD/97/LEM3472	Sudan/Gedaref	MON-267	PKDL	SD/ET1
LEM3429 (LG24)	MHOM/SD/97/LEM3429	Sudan/Gedaref	MON-257	VL	SD/ET1
LEM3463 (LG25)	MHOM/SD/97/LEM3463	Sudan/Gedaref	MON-258	VL	SD/ET1
DEVI (LG9)	MHOM/IN/00/DEVI	India/Bihar	MON-2	VL	IN1
THAK35 (LG10)	MHOM/IN/96/THAK35	India/Bihar	MON-2	VL	IN1
L59	MHOM/LK/2002/L59	Sri Lanka	MON-37	CL	LK
L60c	MHOM/LK/2002/L60c	Sri Lanka	MON-37	CL	LK
L60b	MHOM/LK/2002/L60b	Sri Lanka	MON-37	CL	LK
LEM4537	MHOM/IN/2003/LEM4537	India	MON-37	CL	IN3
LEM4527	MHOM/IN/2003/LEM4527	India	MON-37	CL	IN3
Chandigarh	MHOM/IN/83/Chandigarh	India/Himachal Pradesh	MON-37	VL	IN3
NLB 189	MHOM/KE/83/NLB 189	Kenya	MON37	PKDL	KE/IN2
NLB 323	MHOM/KE/85/NLB 323	Kenya	MON37	VL	KE/IN2
LRC-L57	IMAR/KE/62/LRC-L57	Kenya	MON37	Sandfly	KE/IN2
SC23 (LG16)	MHOM/IN/54/SC23	India	MON-38	VL	KE/IN2
EP3	MHOM/TR/94/EP3	Turkey	n.d.	n.d.	GR/TR MON-1
EP16	MCAN/TR/96/EP16	Turkey	n.d.	CanL	GR/TR MON-1
GH1	MHOM/GR/2001/GH1	Greece/Athens	MON1	VL	GR/TR MON-1
GH3	MHOM/GR/2001/GH3	Greece/Heraklion	MON1	VL	GR/TR MON-1
GH9	MHOM/GR/2001/GH9	Greece/Athens	MON1	VL	GR/TR MON-1
GD3	MCAN/GR/2001/GD3	Greece/Heraklion	MON98	CanL	GR/TR MON-1
GD4	MCAN/GR/2001/GD4	Greece/Heraklion	MON98	CanL	GR/TR MON-1
GH2	MHOM/GR/2001/GH2	Greece/Athens	MON1	VL	GR/TR MON-1
GH5	MHOM/GR/2001/GH5	Greece/Ag.Nikolaos	MON1	VL	GR/TR MON-1
GH6	MHOM/GR/2001/GH6	Greece/Athens	MON98	VL	GR/TR MON-1
GH10	MHOM/GR/2001/GH10	Greece/Athens	MON1	VL	GR/TR MON-1
GH11	MHOM/GR/2001/GH11	Greece/Athens	MON1	VL	GR/TR MON-1
GH12	MHOM/GR/2002/GH12	Greece/Ziros	MON1	VL	GR/TR MON-1
GD7	MCAN/GR/2001/GD7	Greece/Heraklion	MON1	CanL	GR/TR MON-1
GD8	MCAN/GR/2001/GD8	Greece/Rethymno	MON98	CanL	GR/TR MON-1
L4	MHOM/GR/78/L4	Greece/Athens	MON1	VL	SP/PT MON-1 (Spain, Portugal)
IMT337	MHOM/PT/2003/IMT337	Portugal/Alto Douro	MON1	CL	SP/PT MON-1 (Spain, Portugal)
IMT296	MHOM/PT/2002/IMT296	Portugal/Lisbon-MRL	MON1	VL (HIV+)	SP/PT MON-1 (Spain, Portugal)
BCN16 (LG5)	MHOM/ES/86/BCN16	Spain/Catalonia	MON1	CL	SP/PT MON-1 (Spain, Portugal)
IMT260 (LG6)	MHOM/PT/2000/IMT260	Portugal/Lisbon-MRL	MON1	CL	SP/PT MON-1 (Spain, Portugal)
LLM-1136	MCAN/ES/2001/LLM-1136	Spain/Madrid	MON1	CanL	SP/PT MON-1 (Spain, Portugal)
LLM-1106	MCAN/ES/2001/LLM-1106	Spain/Madrid	MON1	CanL	SP/PT MON-1 (Spain, Portugal)
LEM75 (LG1)	MHOM/FR/78/LEM75	France	MON1	VL	SP/PT MON-1 (Spain, Portugal)
LSL29 (LG4)	MHOM/FR/97/LSL29	France	MON1	CL	SP/PT MON-1 (Spain, Portugal)
PM1 (LG3)	MHOM/ES/93/PM1	Spain/Majorca	MON1	VL	SP/PT MON-1 (Spain, Portugal)
LLM-1155	MCAN/ES/2002/LLM-1155	Spain/Ibiza	MON1	CanL	SP/PT MON-1 (Spain, Portugal)
LLM-1233	MCAN/ES/2003/LLM-1233	Spain/Ibiza	MON1	CanL	SP/PT MON-1 (Spain, Portugal)
LLM-1228	MCAN/ES/2003/LLM-1228	Spain/Ibiza	MON1	CanL	SP/PT MON-1 (Spain, Portugal)
LLM-1241	MCAN/ES/2003/LLM-1241	Spain/Ibiza	MON1	CanL	SP/PT MON-1 (Spain, Portugal)
LLM-1203	MCAN/ES/2002/LLM-1203	Spain/Ibiza	MON1	CanL	SP/PT MON-1 (Spain, Portugal)
LLM-1038	MCAN/ES/2001/LLM-1038	Spain/Majorca	MON1	CanL	SP/PT MON-1 (Spain, Portugal)

The non MON-1 strains from Turkey as well as the Cyprian canine isolate clone 1 analysed herein are presented in bold.

VL, visceral leishmaniasis; CL, cutaneous leishmaniasis; PKDL, post Kala Azar dermal leishmaniasis; CanL, canine leishmaniasis.

*Only one of the three MON-37 clones (cl.1) isolated from the parent CD44 strain was further analyzed;

**Hybrid strain; CY, Cyprus; ET, Ethiopia; GR, Greece; IN, India; KE, Kenya; LK, Sri Lanka; PT, Portugal; SD, Sudan; SP, Spain; TR, Turkey; n.d., not defined.

The microsatellite profiles and genetic groups of 68 previously analyzed strains belonging to the major *L. donovani* populations, the non MON-1 *L. infantum* population and the MON-1 population have been described in previous publications [16], [17], [18], [21]. These *L. infantum* and *L. donovani* strains were included for comparison because they originate from various geographic regions and reflect the zymodeme diversity of the two species.

The strains were grown as promastigotes at 26°C in RPMI 1640 containing 20 µmol/L HEPES buffer (GIBCO-BRL Paisley, UK), supplemented with 2 mmol/L glutamine, 10% heat-inactivated fetal bovine serum, 100 IU/mL penicillin, and 100 µg/mL streptomycin.

Total parasite DNA was extracted from mass cultures or clinical samples using phenol/chloroform [22], suspended in water, and stored at −20°C. DNA was isolated from stocks that in their majority were subjected to limited *in vitro* passages.

### Parasite cloning

Based on previous preliminary results [12] we decided to clone, strain CD44 isolated in 2005 from a dog in Cyprus (unpublished data, Gouzelou et al.), and EP59 isolated in 2001 from a VL patient in south-west Turkey. Cloning of *Leishmania* promastigotes from cultures was carried out by the hanging drop method [23]. In brief, single parasites (examined under the microscope independently by two researchers for confirmation) were isolated from minute drops of serially diluted parasite cultures, grown in 96-well plates in RPMI and subsequently on Novy-MacNeal-Nicolle (NNN) [23], [24].

### *K26*-PCR analysis

The *K26*-PCR assay is specific to the *L. donovani* complex and is capable of discriminating *L. infantum* MON-1 from other *L. donovani* complex subspecies, based on length polymorphism of *K26* gene. This assay was applied on clones of CD44, the CUK isolates as well as on parent and clones of the EP59 strains. Amplification reactions and subsequent determination of the amplicon size were carried out as described previously [25].

### MLMT and data analysis

Fourteen variable microsatellite markers randomly distributed throughout the genome (Li 22-35, Li 23-41, Li 41-56, Li 45-24, Li 46-67, Li 71-5/2, Li 71-7, Li 71-33, Lm2TG, Lm4TA, TubCA, CS20, kLIST 7031, kLIST 7039) were used in the present study as previously described [21]. For the amplification of a microsatellite locus, one of each pair of the HPLC-purified primers (Proligo, France) used, was conjugated at its 5′ end to one of three fluorescent dyes. PCR reactions and amplifications were performed using the PTC-200 thermocycler (MJ Research Inc., Watertown, MA) as described elsewhere [21], [26]. The amplified fragments were subjected to automated fragment analysis on the capillary sequencer (CEQ 8000; Beckman Coulter) and analysed with the AFLP analysis software.

To infer the population structure of the sample set, the multilocus microsatellite genotypes were analysed using a model-based clustering method implemented in STRUCTURE v 2.3.1 [27]. This algorithm can assign individuals to populations probabilitistically, based on their multilocus genotypes, and can estimate the posterior probability for a given number of genetic populations (K) thus enabling the identification of the most likely number of populations. The admixture model with correlated allele frequencies was assumed and a burn-in time of 20,000 followed by 200,000 iterations was used. Ten independent runs for each K were carried out for each possible number of clusters (*K*) in order to quantify the variation in the likelihood of the data for a given *K*.

The most probable number of genetic clusters was estimated by comparing log-likelihood values for K between 1 and 12. The major population structure was captured at the plateau (maximum) of the derived Gaussian graph. In addition, as suggested by Evanno et al. to better determine the number of populations, ΔK was calculated for each K [28], which is based on the second-order rate of change in the log probability of data with respect to successive K values.

Microsatellite-based genetic distances were calculated using the software Microsat [29] by applying the proportion of shared alleles (Dps) distance measure [30]. Dps is defined as the negative logarithm of the proportion of shared alleles and follows the infinite allele model (IAM). On the basis of the calculated distance matrices, a midpoint-rooted neighbour-joining (NJ) tree was constructed using PHYLIP, v 3.6 [31]. Confidence intervals were obtained by bootstrapping (1000 replicates). For the construction of a consensus tree and additional tree editing the programs Geneious [32] and Figtree (http://tree.bio.ed.ac.uk/software/figtree) were utilized.

To further characterise the genetic substructure at both population and individual level, a factorial correspondence analysis (FCA) implemented in GENETIX v 4.03 software [33] was carried out. This analysis places the individuals in three-dimensional space according to the degree of their allelic state similarities.

In order to analyse the populations defined by STRUCTURE with respect to diversity of alleles (A), expected heterozygosity (He), observed heterozygosity (Ho) and inbreeding coefficient F_IS_, the GDA software package was applied (http://hydrodictyon.eeb.uconn.edu/people/plewis/software.php).

### Ethical considerations

Strain collection and *Leishmania* DNA used in this study, as well as the respective ethical approvals, were described in previous publications [12], [16], [17], [18], [19], [21]. All samples were anonymized. The present study was approved by the Ethics Committee of the Hellenic Pasteur Institute.

## Results

### *K26*-PCR & MLEE analysis

**Figure 1:**
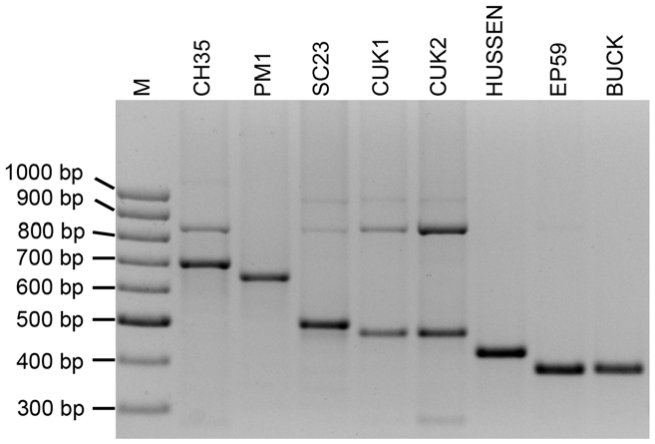
*K26*-PCR confirms that CUK1, CUK2 and EP59 strains belong to the *L. donovani* complex. Lanes: M, 100 bp DNA ladder; CH35 (MON-37, 700 bp); PM1 (MON-1, 626 bp); SC23 (MON-38, 515 bp); CUK1 (MON-309, 480 bp); CUK2 (MON-309, 480 bp); HUSSEN (MON-31, 430 bp); EP59 (MON-308, 385 bp); BUCK (MON-78, 385 bp). Strains CUK3, CUK4, CUK7 and CUK 10 gave identical results to CUK1 and CUK2 (not shown).

The *K26*-PCR assay was the method of choice for initially characterizing the Turkish human and sand fly isolates from the CL foci in Çukurova as well as the VL isolate from Kuşadasi. A *K26* amplicon of 385 bp was obtained for the EP59 strain and its clones. The same size for the *K26* amplicon has been previously observed in the MHOM/MT/85/BUCK strain [25], an *L. infantum* MON-78 strain from Malta that was also included in our analysis for comparison (Table 1, Fig. 1). All 6 strains from the Çukurova region presented a 480 bp *K26* amplicon, which was observed for the first time. The isolates from Cyprus including the MON-37 strain (CH35) used as reference herein gave a 700 bp amplicon [12]. A 626 bp *K26* amplicon typical for strains of zymodeme MON-1 was yielded for the PMI strain from Spain as expected (Fig. 1).

Subsequent MLEE analysis of four of the Turkish isolates revealed two novel *L. donovani* zymodemes. The EP59 strain was typed as MON-308 with enzymatic patterns identical to those of *L. infantum* MON-1 except for the two glutamate-oxaloacetate transaminases (GOT_1_ and GOT_2_, EC 2.6.1.1). In particular, iso-electrofocusing showed a heterozygous pattern between the *L. infantum* GOT^100^ and the *L. donovani* GOT^113^ (F. Pratlong, unpublished data). The three isolates from Çukurova, namely CUK1, CUK2 and CUK3 presented exactly the same enzymatic patterns and were typed as *L. donovani* MON-309. This variant had identical isoenzyme electrophoretic mobilities with the *L. donovani* MON-3 zymodeme apart from malate dehydrogenase (MDH, EC 1.1.1.37) that presented a relative mobility of 145. This is distinct from all other known electrophoretic variants of MDH found among *L. donovani* complex strains. Compared to the MON-37 zymodeme, the novel MON-309 zymodeme differs in the mobilities of just two isoenzymes, notably MDH and glucose phosphate isomerase (GPI, EC 5.3.1.9).

### MLMT analysis

Using a set of 14 high-resolution microsatellite *L. donovani* complex specific markers [26] all newly analyzed Turkish strains (TR) presented unique genotypes and significant intra-zymodeme diversity was detected. Among strains of the newly identified MON-309 zymodeme different levels of allelic variation were observed that range from a single difference in 1 allele (strains CUK1 and CUK2) to variations in up to 8 alleles (strains CUK2 and CUK10). Interestingly the EP59 strain was characterised by an unusual high number of heterozygous loci (9 out of 14 loci studied). In each of these loci one allele was typical MON-1 and the second corresponded to the allele sizes found for the CUK strains (Table S1).

In order to infer the population structure/substructure of our sample set the MLMT profiles of the MON-308 and MON-309 strains as well as that of the CD44 MON-37 clones (Table S1) were compared to those of previously typed *L. infantum* and *L. donovani* strains of different zymodemes and different geographical origins (Table 1) [16], [17], [18], [21]. The population structure of the sample set was estimated using various methods, each one with different assumptions.

**Figure 2:**
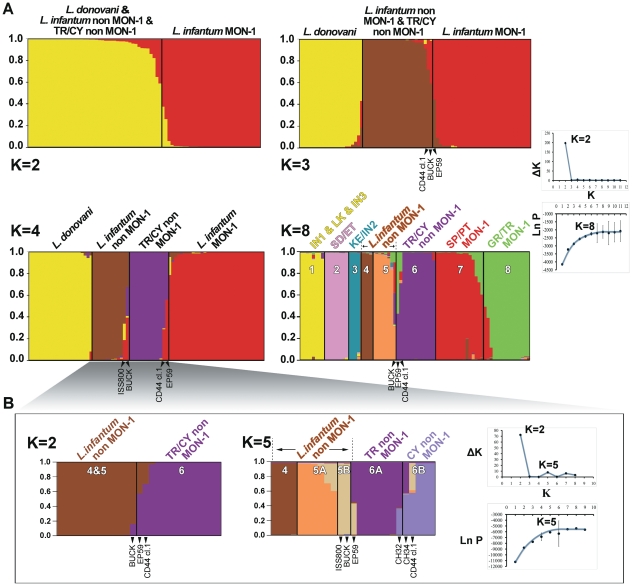
Estimated population structure of the 76 *L. donovani s.l.* strains as inferred by STRUCTURE. MLMT profiles are based on variations in 14 microsatellite markers. Each strain is represented by a single vertical line divided into K colours, where K is the number of populations assumed. Each colour represents one population and the length of the coloured segments shows the strain's estimated proportion of membership in that population. Strains with mixed memberships to the different populations are represented by different coloured segments in the vertical bar, which are proportional to the membership coefficient. **A.** When the likelihood of population number is calculated according to Evanno et al. [28], the derived graph for ΔK shows a peak at K = 2 indicating the existence of two main populations in the studied strain set. However, eight populations are observed based on a log-likelihood plot, which plateaus at K = 8. At K = 8, all Turkish non MON-1 strains group with MON-37 strains from Cyprus, forming the TR/CY non MON-1 population. Bar plots for K = 3 and K = 4 are also shown to help determine ancestral populations. **B.** When the *L. infantum* non MON-1 & TR/CY non MON-1 strains are re-analysed separately the log-likelihood plot plateaus at K = 5, while the ΔΚ graph shows a major peak at K = 2 and a minor one at K = 5. At K = 5 a split of the TR/CY non MON-1 population was observed.

Firstly, we employed the Bayesian genotype clustering program STRUCTURE v 2.3.1 [27]. When the K estimator derived from the second-order rate of change of the likelihood function with respect to K [28] was examined, a sharp signal was found at K = 2 (Fig. 2A). This suggested that two major homogeneous gene pools shape the genetic structure of the analysed strains. At K = 2, MON-1 strains form the first population and all non MON-1 strains, the previously characterized *L. donovani* strains and *L. infantum* non MON-1 as well as those from Turkey (TR), were grouped in the second ancestral source population (Fig. 2A). At K = 3, the *L. donovani* population splits to form a distinct one (Fig. 2A). Interestingly, at K = 4 the 6 strains from Çukurova region and the recently identified MON-37 isolates from Cyprus (CY) [12], [13], [16] group into a separate population, hereafter termed TR/CY non MON-1. This population also included one of the MON-37 clones of the CD44 canine isolate (CD44 cl.1). The EP59 strain could not be assigned to only one population but had shared membership to both the GR/TR MON-1 (Greece/Turkey) and TR/CY non MON-1 populations (Fig. 2A).

It is evident that in the previous estimation the uppermost hierarchical level of population structure was captured. However, the logarithm of probability of the data reached a plateau at K = 8 revealing the presence of additional subpopulations (Fig. 2A). All populations were well defined and stable at K = 8, and corresponded generally to the geographical origin of the strains. The eight main populations were: (1) IN1 (India 1), IN3 (India 3) and LK (Sri Lanka); (2) SD/ET (Sudan/Ethiopia); (3) KE/IN2 (Kenya/India 2); (4) *L. infantum* non MON-1 population 1; (5) *L. infantum* non MON-1 population 2; (6) TR/CY non MON-1 (Turkey/Cyprus); (7) SP/PT MON-1 (Spain/Portugal); and (8) GR/TR MON-1 (Greece/Turkey). The main *L. donovani* complex genetic populations have been previously identified in various studies [16], [17], [18], [21] and were also apparent in our analysis. Similarly, populations IN1, LK, IN3 have been analysed extensively in other publications [16], [21] and were also well-defined in our analysis when larger K values (>10) were estimated (not shown). The TR/CY non MON-1 population is maintained without change from K = 4 to K = 9. However, at K = 10 it splits further to form the TR non MON-1 and the CY non MON-1 populations (not shown).

As suggested by Evanno et al. [28], in order to find the hidden sub-group structure, the ancestral group subsets defined by the program should be reanalysed independently. As defined at K = 3 (Fig. 2A), the analysis was repeated only for the ancestral non MON-1 population, which is comprised of *L. infantum* non MON-1 and TR/CY non MON-1 and excluded the well characterized *L. donovani* and *L. infantum* MON-1 populations. In the new analysis a strong ΔK peak was observed at K = 2 and a weaker one at K = 5 (Fig. 2B). In addition, K = 5 was the most likely number of populations when the logarithm of probability of the data was plotted with respect to K. At K = 2, *L. infantum* non MON-1 and TR/CY non MON-1 were the two observed populations while at K = 5 the TR/CY non MON-1 population splits into the TR non MON-1 and CY non MON-1 populations (Fig. 2B). At this split the Cypriot CH32 strain (Table 1) groups with the Turkish isolates and the CH34 strain shows a significant proportion of membership to the TR non MON-1 population (Fig. 2B). The fact that similar results were observed when analysing all strains together and when focusing only on the non MON-1 strains corroborates this analysis to a great extent.

**Figure 3 pntd-0001507-g003:**
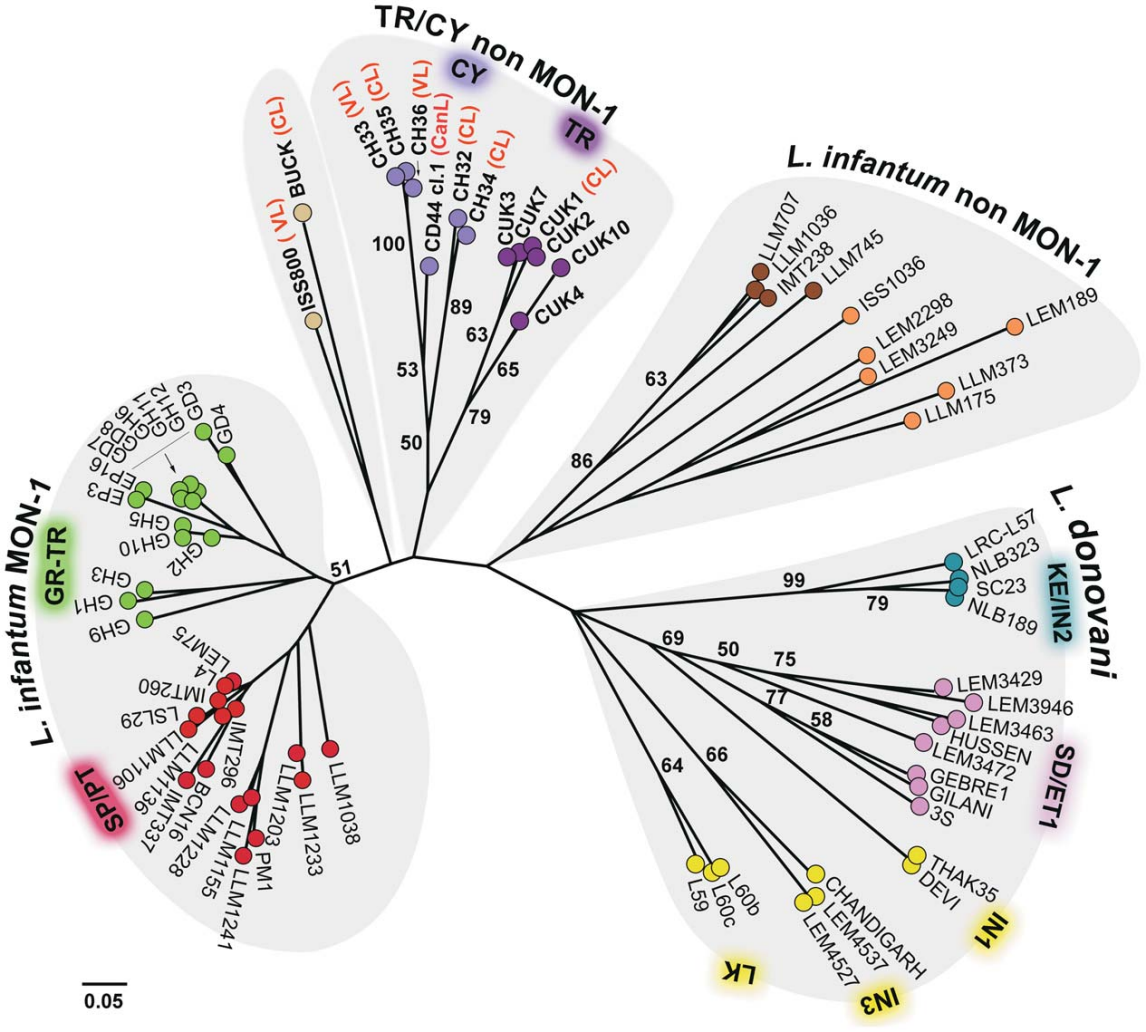
Midpoint rooted Neighbor-joining tree for the 76 *L. donovani* complex strains analysed. This midpoint rooted tree was inferred from Dps-distances calculated for the MLMT data (14 microsatellite markers) of our sample set. The tree leaves are coloured according to the populations defined by structure analysis (Fig. 2). Bootstrap values (1000 re-samplings) above 50% are indicated at key nodes. The EP59 strain was excluded from this analysis due to its hybrid profile.

Secondly, we applied the proportion of shared alleles (Dps) distance measure [30] to estimate population differentiation under the assumption of an Infinite Allele Model (IAM) mutation model. The matrix was calculated using the microsatellite profiles of all strains under study (Table 1), excluding the EP59 strain to avoid a false phylogeny due to its ‘hybrid’ profile. The generated midpoint rooted NJ tree is shown in Figure 3. Four main clusters are observed corresponding to the 4 populations obtained by STRUCTURE at K = 4. Furthermore, in accordance with STRUCTURE analysis (Fig. 2B, K = 5) the TR/CY non MON-1 cluster is formed by two geographically defined subclusters, one containing all Turkish strains (TR non MON-1) and the other containing the MON-37 Cypriot strains (CY non MON-1). Interestingly, the TR/CY non MON-1 clade is placed between a small group, which includes the strains MHOM/MT/85/BUCK, MHOM/IT/93/ISS800, and the *L. infantum* non MON-1 clade. Confidence intervals were obtained by bootstrapping (1000 replicates). Although bootstrap support of >50% was observed between most subclusters, including that of the studied TR non MON-1 strains, it was weak for the basal nodes discriminating the major clusters.

**Figure 4:**
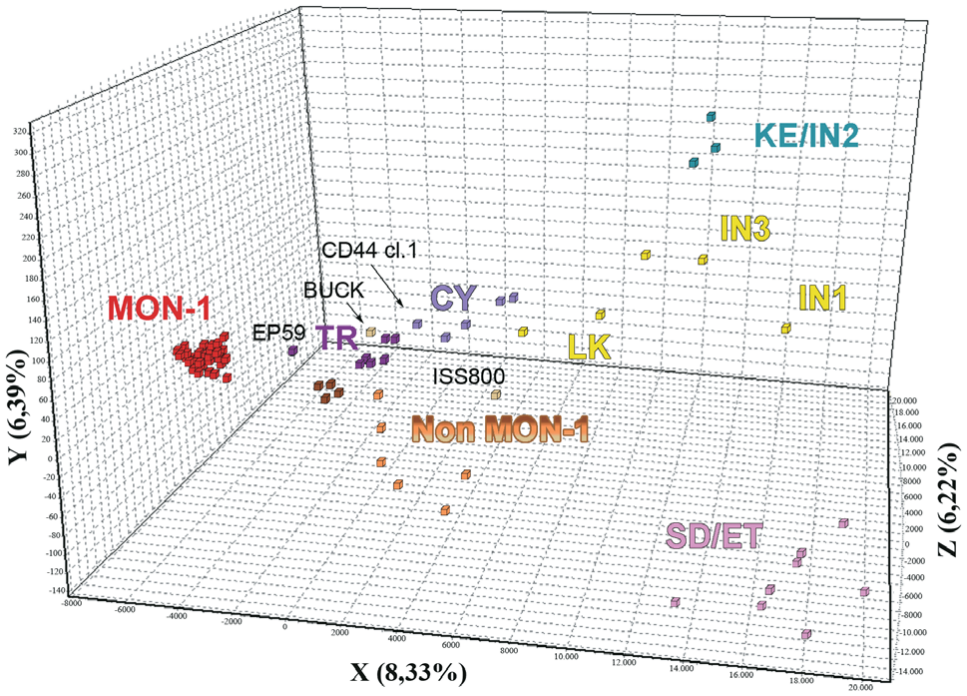
Factorial correspondence analysis (FCA) of the 76 *L. donovani* complex strains studied. CY, Cyprus; ET, Ethiopia; GR, Greece; IN, India; KE, Kenya; LK, Sri Lanka; PT, Portugal; SD, Sudan; SP, Spain; TR, Turkey; Populations designated as MON-1 (red squares) and non MON-1 (orange and brown squares) include respectively *L. infantum* MON-1 strains from Greece, Turkey, Spain and Portugal and *L. infantum* non MON-1 strains from Spain, Portugal, France and Italy, corresponding to previous analyses [18], [21]; populations designated as IN1 (India 1), IN3 (India 3) and LK (Sri Lanka) (yellow squares); SD/ET (Sudan/Ethiopia) (pink squares); KE/IN2 (Kenya/India 2) (blue squares) compose the *L. donovani* genetic group, as detected previously [16], [17], [18], [21]; *L. infantum* non MON-1 strains from Turkey and Cyprus are designated as TR (dark purple squares) and CY (light purple squares), respectively. The MON-37 clone of CD44 strain from Cyprus groups with the other CY non MON-1 strains. The TR/CY strains are placed between the *L. infantum* MON-1 and non MON-1 populations and the hybrid strain EP59 from Turkey between the TR non MON-1 and MON-1 populations. The BUCK strain is placed very close to the TR non MON-1 strains, as previously described [18], [21].

Factorial correspondence analysis (FCA) of the MLMT data corroborated our observations concerning the genetic relationships among the strains in our sample set and particularly the existence of a distinct population containing the strains from Turkey and Cyprus. The TR/CY strains were placed in an intermediate position between the MON-1 and the *L. infantum* non MON-1 populations (Fig. 4). Interestingly, the EP59 strain did not tightly group to the other TR non MON-1 strains but had a shifted position towards the MON-1 population, between the TR non MON-1 and the MON-1 populations. In addition, the BUCK strain (Table 1) was placed very close to the TR non MON-1 strains (Fig. 4) confirming previously described analyses.

### Population structure

The expected heterozygosity (*He*), as a measure of genetic diversity, was higher in the TR/CY non MON-1 strains (0.518) compared to MON-1 (0.276 for SP/PT, and 0.246 for GR/TR) strains (Table 2). The TR/CY non MON-1 population's high genetic diversity was in fact comparable to that of the other *L. infantum* non MON-1 strain population (0.710). This was also apparent from the greater genetic distances on the Neighbor-joining tree (Fig. 3).

**Table 2:** Genetic variability parameters of the strain populations under study.

Population	N	P	MNA	H_e_	H_o_	F_IS_
**K = 8 (** **Fig. 2A** **)** *						
SP/PT MON-1	16	0.714	2.57	0.276	0.031	0.890
GR/TR MON-1	15	0.571	2.00	0.246	0.019	0.925
*L. infantum* non MON-1^a^	12	1.000	5.43	0.710	0.196	0.732
TR/CY non MON-1	12 (13)^b^	0.929 (1.000)^b^	3.29 (2.86)^b^	0.518 (0.533)^b^	0.180 (0.217)^b^	0.662 (0.604)^b^
**K = 5 (** **Fig. 2B** **)** **						
TR non MON-1	6 (7)^b^	0.571 (0.786)^b^	1.64 (2.21)^b^	0.258 (0.325)^b^	0.136 (0.211)^b^	0.498 (0.370)^b^
CY non MON-1	6	0.857	2.29	0.365	0.226	0.403

N, number of strains; P, proportion of polymorphic loci; MNA, mean number of alleles; H_e_, expected heterozygosity; H_o_, observed heterozygosity; F_IS_, inbreeding coefficient; CY, Cyprus; GR, Greece; PT, Portugal; SP, Spain; TR, Turkey.

*When all 76 strains are analyzed 8 populations are identified by STRUCTURE (Fig. 2A). Here the genetic variability parameters of four of these populations are shown;

a The two *L. infantum* non MON-1 populations are analyzed together;

**At a subsequent STRUCTURE analysis the TR/CY non MON-1 group splits into two subpopulations (TR non MON-1 and CY non MON-1, Fig. 2B), which are analyzed here;

b The respective values when the hybrid EP59 strain is included in the respective population are given in parenthesis.

Either a single or two different alleles were observed for all strains in all the loci under study. More than two peaks, suggestive of aneuploidy or mixed heterozygous strains, did not occur. For all populations, the observed heterozygosity values were significantly lower than the expected ones and were higher in *L. infantum* non MON-1 (0.196) and TR/CY non MON-1 (0.180) strains compared to *L. infantum* MON-1 strains (0.031 for SP/PT and 0.019 for GR/TR) (Table 2).

The inbreeding coefficients of the TR/CY non MON-1 population was calculated at 0.662, the lowest value amongst the 4 populations studied here (*F_IS_* 0.890 for SP/PT MON-1, 0.925 for GR/TR MON-1 and 0.732 for *L. infantum* non MON-1). The highly positive *F_IS_* values can be due to the presence of different factors, such as population subdivision (Wahlund effect) or a high rate of gene conversion.

### Indications for recombination and mixed infections

The EP59 strain had an unusual high number of heterozygous loci with alleles characteristic for both the GR/TR MON-1 and TR/CY non MON-1 populations and was assigned to intermediate positions between these two populations (Fig. 1, 2 and 4). Because this suggested that the EP59 strain might represent either mixed MON-1 and MON-308 strains or a MON-1/MON-308 hybrid we decided to clone EP59. Four clones were obtained (EP59 cl1 to EP59 cl4) and re-analysed by MLMT. Interestingly, all clones had identical MLMT profiles to the uncloned strain suggesting that it might be a hybrid (Table S1).

We have also analysed one out of the three MON-37 CD44 clones, isolated in 2005 from a dog in Cyprus [12]. The three CD44 MON-37 clones (CD44cl.1-CD44cl.3) gave the same size for the K26 amplicon (700 bp) as the MON-37 strains whereas two other clones (CD44cl.4 and CD44cl.5) gave an amplicon typical for MON-1 (data not shown). Also, CD44 clones 1–3 presented identical microsatellite profiles and had allele sizes common to those of the MON-37 Cypriot population (CY) [12], [16]. Interestingly these clones were heterozygous at 6 out of 14 loci studied (Table S1). This result confirmed preliminary MLMT data suggesting that the dog was co-infected with both MON-1 and MON-37 strains [12].

## Discussion

In this study, we seek to further analyse strains of the *L. donovani* complex, isolated in Turkey from human CL foci in Çukurova (CUK strains) and from one human VL case in Kuşadasi (strain EP59), and to compare them with strains from Cyprus (CH strains and the MON-37 CD44 clones). These Cypriot strains were typed previously as *L. donovani* MON-37 by MLEE but shown to be genetically distinct from the MON-37 strains of other regions [16]. In line with previous DNA-based data [6], [19], using the *K26*-PCR assay, the Turkish strains were inferred to the *L. donovani* complex. Interestingly, they did not present the *K26* amplicon sizes corresponding to the *L. infantum* MON-1 zymodeme [25] and were thus characterized as non MON-1. Subsequent MLEE analysis of four of the Turkish isolates revealed two novel *L. donovani* zymodemes, namely zymodemes MON-308 and MON-309. The Turkish strains from Çukurova (zymodeme MON-309) and the MON-37 strains from Cyprus differed in just two isoenzymes and hence MLEE suggested a close relationship between them. Notably, when the EP59, CUK2 and CH35 strains (a MON-37 strain from Cyprus) were subjected to PCR-RFLP of the *cpb*EF gene [34] an *L. donovani* profile was revealed (data not shown).

Overall our data point out the need to validate PCR typing assays on a geographically representative panel of isolates for a given region and to account not only for intra-species variability but also for species identification. This applies especially to areas where different sympatric species are present, as in the EMR. Also we would like to emphasize that for regions with relatively limited data on species distribution of the so called *L. donovani* complex, the specificity of the PCR tests should be validated by including a sufficient number of reference strains that fall in the boundaries between *L. donovani* and *L. infantum* species. Otherwise DNA-based typing could be erroneous. The *K26*-PCR assay is a simple and quick method that could prove valuable for assessing simultaneously whether the causative agent of VL or CL belongs to the *L. donovani* complex and if it is a MON-1 zymodeme.

The MLMT approach that is highly discriminatory and reproducible was chosen for accurately investigating the genetic diversity and population structure of the Turkish strains and for classifying them within the *L. donovani* complex. Considerable polymorphism, comparable to that observed within the *L. infantum* non MON-1 group was detected within the studied Turkish strains. This is demonstrated by higher *He* and *Ho* values (Table 2), long branches in the N-J tree (Fig. 3) and a broad distribution of the strains in FCA (Fig. 4).

The most striking outcome of the MLMT analysis was the identification of a ‘new’ main cluster within the *L. donovani* complex, proposing the existence of four main *L. donovani* complex populations. All non MON-1 Turkish isolates were grouped with the recently isolated MON-37 strains from Cyprus [12], [13] in a single monophyletic group (TR/CY non MON-1) of close relationship to the *L. infantum* MON-1 group (Fig. 2–4). The remaining three clusters, namely *L. infantum* MON-1, *L. infantum* non MON-1 and *L. donovani*, are in agreement with previous publications [16], [17], [18], [21]. The fact that the same main populations were identified when both model- and distance-based methods were applied for the analysis of the microsatellite data, confirms the validity of our analysis. The main subclusters in the N-J tree were also supported by bootstrap values greater than 50%. Basal notes of the major clusters had weak support but this is commonly observed because microsatellite data become less informative for distantly related taxa [30].

Furthermore, strains BUCK and ISS800 formed an additional small cluster at the base of MON-1 and TR/CY non MON-1 (Fig. 3). The strains also grouped in a subpopulation at K = 5 when analysed with STRUCTURE (Fig. 2B) and BUCK had a significant membership to the TR/CY non MON-1 population at K = 8 (Fig. 2A). Grouping of ISS800 with CUK strains was also demonstrated by Svobodova et al. [6]. Several studies using different genetic markers have placed these two strains (BUCK and ISS800) in ambiguous intermediate positions. Specifically, the strains were either assigned at the basis of the non MON-1 group right after the split from *L. donovani* [21], [35] or close to the MON-1 group [18], [36], as observed in our analysis. The inclusion of the TR/CY non MON-1 strains seems to better clarify the ambiguous position of the two strains but still a larger set of strains should be analysed to reach a solid conclusion.

Interestingly, in a previous work where the same strains from Cyprus were analyzed [16] it was shown that the MON-37 zymodeme is paraphyletic, encompassing strains with the same enzymatic profile but with different genotypes and consequently their origin still remained enigmatic. Here, we show that the Cypriot strains are very closely related to the Turkish strains. Therefore, the strains could have potentially been introduced into Cyprus from Turkey or vice versa. The geographic proximity of the countries and population movement between them further supports this scenario. Nevertheless, the possibility that these strains originate from neighbouring Middle East countries cannot be excluded.

Parasites isolated from both human hosts (EP59, CUK1) and *P. (Larroussius) tobbi* sand fly vectors (CUK2, CUK3, CUK4, CUK7 and CUK10) clustered in the same MLMT groups, confirming that *P. (Larroussius) tobbi* is responsible for the transmission of these parasites in the Çukurova focus [6]. Apart from the proven vector *P. (Larroussius) tobbi*, other members of subgenus *Larroussius* could facilitate circulation of TR/CY non MON-1 parasites. Sand flies of this subgenus belong to permissive vectors [37] and are widely distributed throughout Middle East countries, including Cyprus, Greece and Turkey [6], [13], [38], [39]. *P. (Larroussius) perniciosus*, a major *L. infantum* vector in the central and western Mediterranean, was recently shown to be highly susceptible for CUK strains and could readily transmit the CUK parasites by bite [40]. In Iran, six *Leishmania* strains isolated from *P. (Larroussius) perfiliewi transcaucasicus* were typed as *L. donovani* by *cpb* gene sequencing [11].

Notably, the strains from Çukurova were typed as *L. donovani* by MLEE, even though in our MLMT analysis they group between the *L. infantum* MON-1 and non MON-1 populations (Fig. 2 and 3). Discrepancies between MLEE typing and genotyping especially regarding the taxonomy of the “*L. donovani*” complex have already been described in a number of studies [2], [36], [41], [42]. This is not so surprising since MLEE mainly distinguishes the species of the *L. donovani* complex on the basis of just the gene encoding glutamate-oxaloacetate transaminases (GOT) isoenzymes, EC 2.6.1.1, which has been shown to have undergone convergent evolution [43].

Interestingly the putative hybrid EP59 strain was responsible for VL in a human patient from Western Turkey (Kuşadasi). This intra-specific putative hybrid is most likely a product of genetic cross between strains of the MON-1 and the non MON-1 TR/CY populations. The occurrence of genetic exchange in the genus *Leishmania* had remained an elusive point until recently, when Akopyants et al. provided evidence that *Leishmania* promastigotes are capable of having a sexual cycle consistent with a meiotic process in the sand fly [44]. Also Sadlova et al. demonstrated experimentally hybridization of *L. donovani* in two vectorial species, *P. perniciosus* and *Lutzomyia longipalpis* [45]. *Leishmania* hybrids have already been identified between closely related species, such as *L. braziliensis*/*L. panamensis* [46], [47] and *L. major*/*L. arabica* [48], [49]. Intra-species *Leishmania* hybrids have also been observed in Algeria [50], Tunisia [51], Sudan [2] and Ethiopia [52]. Strains with mixed genotypes are also frequently observed [16], [18]. Here, mixed genotypes were observed for strains CH32 and CH34, which presented alleles of both TR non MON-1 and CY non MON-1 subpopulations (Fig. 2B, K = 5). Furthermore, the canine MON-37 clone from Cyprus (CD44 cl.1) had a shared membership to both the *L. infantum* non MON-1 and the CY non MON-1 populations (Fig. 2B, K = 5).

Hybridization may allow adaptation to new ecological niches, vectors and hosts, including humans and domestic animals, and the efficient spread of new traits. Volf et al. [53] demonstrated that *L. infantum*/*L. major* hybrids could be transmitted efficiently by *P. papatasi*, a highly specific sand fly vector that has been shown to support only the development of *L. major*. Additionally, Nolder et al. [54] described the emergence and spread of *L. braziliensis*/*L. peruviana* hybrids with increased fitness in Peru. However, it is important to mention that the pathology of leishmaniasis varies and is determined not only by parasite genetics but also by other factors such as host genetics [55].

Parasites belonging to the TR/CY non MON-1 population have been isolated from two distant regions of Turkey, the south-eastern (Çukurova) and south-western (Kuşadası) parts, indicating that these parasites could be broadly distributed in Turkey. In line with this hypothesis, their presence in neighbouring countries also merits to be investigated.

Parasites of the identified TR/CY population could be contributing to the generation of CL in this region. In this case, clinical management of these patients would require more accurate monitoring during drug treatment since these parasites could potentially cause VL, which can be lethal if left untreated. Furthermore, appropriate drug regimes should be administered considering different *Leishmania* species show significant variation in their sensitivity to drugs [56]. Distinguishing different *L. donovani* complex species/subspecies is therefore crucial for prognosis, drug regimes and proper disease control.

In conclusion, our analysis indicates that the epidemiology of leishmaniasis in Turkey is more complicated than originally thought. Here we describe a new *L. donovani sensu lato* non MON-1 group of strains originating from Turkey and Cyprus, which can cause both CL and VL. The identified population is genetically different from all other *L. donovani* complex populations, including the *L. infantum* MON-1 population that is also present in these countries. Interestingly, a putative MON-1/TR/CY non MON-1 hybrid strain and some gene flow between the studied populations were also identified. The results described herein and those of previous studies showing that MLEE and DNA-based approaches for strain typing may lead to discrepant results, further support the call for a revision of *Leishmania* taxonomy [2], [21], [36], [41], [42]. This should be based on gene sequences, which are remarkably congruent and uncontroversial. Nevertheless, our findings are inconsistent with the current nomenclature of the *L. donovani* complex with just two species and argue in favour of recognizing a number of *L. donovani* subspecies.

## Supporting Information

Table S1 **Multilocus microsatellite profiles of strains presenting high heterozygosity.** CUK strains present different levels of allelic variation to a maximum of eight allele differences (strains CUK2 and CUK10). Strain EP59 presents extensive heterozygosity (9 out of 14 loci), sharing one typical MON-1 allele and a second one corresponding to the allele sizes of CUK strains. MON-37 CD44 clones are also highly heterozygous (6 out of 14 loci) sharing common alleles with those of Cypriot MON-37 strains (CH35 strain is shown here for comparison). Identical MLMT profiles are obtained for all EP59 clones (EP59cl.1- EP59cl.4) as well as all MON-37 CD44 clones (CD44cl.1- CD44cl.3); only EP59cl.1 and CD44cl.1 are shown here.
(DOC)
