# Supplementary material for: Multilocus Microsatellite Typing (MLMT) of Strains from Turkey and Cyprus Reveals a Novel Monophyletic L. donovani Sensu Lato Group
Source: PLoS Negl Trop Dis. 2012 Feb 14;6(2):e1507. doi: 10.1371/journal.pntd.0001507 (PMC3279343; doi:10.1371/journal.pntd.0001507)
Supplement: Table S1 — Multilocus microsatellite profiles of strains presenting high heterozygosity. CUK strains present different levels of allelic variation to a maximum of eight allele differences (strains CUK2 and CUK10). Strain EP59 presents extensive heterozygosity (9 out of 14 loci), sharing one typical MON-1 allele and a second one corresponding to the allele sizes of CUK strains. MON-37 CD44 clones are also highly heterozygous (6 out of 14 loci) sharing common alleles with those of Cypriot MON-37 strains (CH35 strain is shown here for comparison). Identical MLMT profiles are obtained for all EP59 clones (EP59cl.1- EP59cl.4) as well as all MON-37 CD44 clones (CD44cl.1- CD44cl.3); only EP59cl.1 and CD44cl.1 are shown here. (DOC) [file pntd.0001507.s001.doc]

**Table S1. Multilocus microsatellite profiles of strains presenting high heterozygosity.**

| **Strain code** | **Lm2TG** | **Lm4TA** | **Li41-56** | **Li46-67** | **Li22-35** | **Li23-41** | **Li45-24** | **Li71-33** | **Li71-5/2** | **Li71-7** | **CS20** | **kLIST7031** | **kLIST7039** | **TubCA** |
| --- | --- | --- | --- | --- | --- | --- | --- | --- | --- | --- | --- | --- | --- | --- |
| **ITOB/TR/2005/CUK10** | 7 | 8 | 7 | 9 | 17 | 22 | 11 | 11 | 9 | 13 | 19 | 10 | 20 | 10 |
| **ITOB/TR/2005/CUK2** | 10 | 8 | 7+10 | 9 | 17 | 21 | 7+11 | 11+13 | 8 | 13 | 19 | 11 | 13+20 | 10 |
| **MHOM/TR/2001/EP59cl.1** | 10+25 | 8+11 | 9+10 | 9 | 12+17 | 16+21 | 7+17 | 11+13 | 9 | 13 | 19 | 11 | 15+20 | 9+10 |
| **MCAN/CY/2005/CD44cl.1** | 9 | 12+14 | 10 | 6+9 | 6 | 11+16 | 16 | 11 | 9 | 8+11 | 12 | 8+11 | 15+20 | 10 |
| **MHOM/CY/2006/CH35** | 9 | 12 | 10 | 6 | 6 | 11 | 14 | 11 | 9 | 8 | 11 | 8 | 20 | 10 |
